# “Most of the cases are very similar.”: Documenting and corroborating conflict-related sexual violence affecting Rohingya refugees

**DOI:** 10.1186/s12889-022-13038-7

**Published:** 2022-04-09

**Authors:** Lindsey Green, Thomas McHale, Ranit Mishori, Linda Kaljee, Shahanoor Akter Chowdhury

**Affiliations:** 1 Present Address: Physicians for Human Rights, Boston, USA; 2 Georgetown University School of Medicine, 3900 Reservoir Road, NW, Washington, DC 20057 USA; 3 Henry Ford Health Systems, Global Health Initiative, 1 Ford Place, Suite E1, Detroit, MI 48202 USA; 4 Physicians for Human Rights, Dhaka, Bangladesh

**Keywords:** Rohingya, Conflict-related sexual violence, Humanitarian response, Human rights, Myanmar

## Abstract

**Background:**

In August 2017, a large population of Rohingya from northern Rakhine state in Myanmar fled to Bangladesh due to “clearance operations” by the Myanmar security forces characterized by widespread and systematic violence, including extensive conflict-related sexual violence (CRSV). This study sought to document the patterns of injuries and conditions experienced by the Rohingya, with a specific focus on sexual violence.

**Methods:**

Qualitative interviews were conducted with 26 health care professionals who cared for Rohingya refugees after their arrival in Bangladesh between November 2019 and August 2020.

**Results:**

Health care workers universally reported hearing accounts and seeing evidence of sexual and gender-based violence committed against Rohingya people of all genders by the Myanmar military and security forces. They observed physical and psychological consequences of such acts against the Rohingya while patients were seeking care. Health care workers shared that patients faced pressure not to disclose their experiences of CRSV, likely resulted in an underreporting of the prevalence of sexual violence. Forced witnessing of sexual violence and observed increases in pregnancy and birth rates as a result of rape are two less-reported issues that emerged from these data.

**Conclusions:**

Healthcare workers corroborated previous reports that the Rohingya experienced CRSV at the hands of the Myanmar military and security forces. Survivors often revealed their experiences of sexual violence while seeking care for a variety of physical and psychological conditions. Stigma, cultural pressure, and trauma created barriers to disclosing experiences of sexual violence and likely resulted in an underreporting of the prevalence of sexual violence. The findings of this research emphasize the importance of offering universal and comprehensive trauma-informed services to all refugees with the presumption of high rates of trauma in this population and many survivors who may never identify themselves as such.

## Background

In August 2017, Myanmar’s armed forces, known as the Tatmadaw, and security forces committed widespread and systematic violence against Rohingya communities in Myanmar’s northern Rakhine state [1]. These “clearance operations” were, in the words of a 2018 United Nations (UN) independent international fact-finding mission report, “brutal and grossly disproportionate,” targeting hundreds of villages and the entire Rohingya population [2].

The violence caused more than 720,000 Rohingya to flee Myanmar for neighboring Bangladesh between August 25, 2017 and July 31, 2019 [3]. Most refugees arrived in the first 3 months of the crisis, creating the world’s largest refugee camp in the district of Cox’s Bazar [3]. As the Rohingya arrived serious human rights violations, including sexual violence, committed during the “clearance operations” were documented [1, 2, 4–12]. A variety of actors found that attacks against Rohingya communities were excessively violent, widespread, methodical, and committed on a massive scale [1, 8]. The UN fact-finding mission stated “[the attacks] constituted crimes against humanity, war crimes, and underlying acts of genocide accompanied by inferences of genocidal intent.” [2].

Documented acts of conflict-related sexual violence (CRSV) included rape, multiple perpetrator rape, mass rape, sexual assault, sexual violence or the threat of sexual violence followed by the killing of victims, forced witnessing, forced nudity, and violence targeting sexual organs [5, 7, 9–11, 13, 14]. While there has been extensive documentation of violence experienced by the Rohingya while in Myanmar, this research has largely focused on collecting the experiences of individual Rohingya survivors. The observations of health care workers who interacted with or treated Rohingya refugees for their injuries, both physical and psychological, have not been systematically captured. Health care workers play an important role in documenting human rights violations of this nature as they can speak to the reported violations and overall patterns of injuries in this population and identify survivors’ ongoing needs. The documentation of this perspective addresses a gap in the current literature on this topic.

The goal of this research was to document patterns of injuries and understand the health conditions of Rohingya refugees, with a particular focus on sexual violence, after their arrival in Bangladesh after August 2017.

## Methods

This qualitative research was composed of 26 semi-structured interviews with health care workers who provided direct care to Rohingya refugees in Bangladesh between August 2017 and August 2020. We defined health care workers broadly, including physicians, nurses, midwives, mental health and psychosocial support workers, case managers, community health care workers, and health volunteers. Health care workers were affiliated with a variety of organizations and worked in a variety of health care settings, all within the refugee camps which are in the Cox’s Bazar district of Bangladesh. Health care workers who had cared for Rohingya patients in Bangladesh any time after August 2017 were included. Healthcare workers who worked in the refugee camps immediately after August 2017 and in 2018 could capture the acute effects of the violence, while those working in the refugee camps after 2018 could speak to both the violence experienced by survivors in Myanmar and the long-term physical and mental impacts of this violence. Data was collected from November 2019 to August 2020. The study was approved by the Institutional Review Board at Georgetown University (GU-HRB-503) and received exemption through the PHR Ethics Review Board.

Health care worker respondents were identified through snowball sampling. Important profiles for study respondents were identified using inclusion criteria (health care workers who had worked with Rohingya patients in Bangladesh any time after August 2017) and emergent findings to ensure sample diversity and that data collected was ultimately responsive to the research objectives [15]. The final sample size was determined by reaching “data saturation” to maximize variability and ensure adequate data to identify themes and patterns [16].

### Data collection and analysis

Data was collected using a semi-structured interview guide and a demographic form to capture respondents’ background and work experience with the Rohingya. The key topics included in the interview guide were the respondents’ professional background and the context of their work with the Rohingya; their experiences treating Rohingya patients and injuries observed due to physical violence; their experiences treating Rohingya patients in relation to sexual and gender-based violence (SGBV); the mental health status they observed in their patients; factors associated with disclosure of SGBV; and challenges in providing health care and addressing trauma. The interview guide topics were consistent throughout the project, however as the team collected and analyzed data, changes were incorporated into the guide to ensure that interviews addressed emergent themes.

All participants completed an informed consent process including a written form and oral confirmation before commencing qualitative interviews. With acknowledgment of the ethical concerns related to sharing patients’ experiences that were shared with health care workers in confidence, respondents were not asked to provide specific patient’s stories but rather speak to their own recollections and experiences. When respondents shared individual patient’s stories, by way of example, they were not asked to provide any identifiable information regarding the patient’s experiences shared as part of the interview.

All interviews except one were conducted remotely via video and voice calls. Each interview took approximately 50 min. Interviews (*n* = 20 in English and *n* = 6 in Bangla) were conducted by four researchers (two physicians and two social scientists based in the United States and Bangladesh). All interviewers received orientation to the research objectives, interview guide and key themes and interview procedures to include a standardized approach. Interviewers participated in frequent debriefs throughout the data collection process to ensure continued alignment on the data collection approach. Interviews conducted in English were transcribed verbatim by a professional transcription service (Rev). Interviews in Bangla were transcribed and translated into English by a team of qualified transcribers and translators and reviewed for accuracy by the interviewer, fluent in both English and Bangla.

All interview transcripts were deidentified prior to analysis and any identifiable information recorded about respondents was saved separately from all data in password protected files.

The research team reviewed the data and developed a coding dictionary. After a first round of coding in Dedoose [17], the team conducted a qualitative intercoder reliability assessment on a sample of transcripts which indicated consistency in overall use of the codes as they were defined for the study.

The research team reviewed coded data, identified themes and patterns, and created a narrative reflecting the data, responding to the research objectives and interpreted within the context of other published sources on sexual and gender-based violence among the Rohingya.

## Results

Twenty-six health care professionals from different countries and different specialties and disciplines were interviewed. Table 1 provides a description of the cohort.

**Table 1:** Demographic information for respondents

Respondents	
*n=*	26
Respondent gender	
*f=*	20
*m=*	6
Respondent age	
*25–34 years old*	11
*35–44 years old*	10
*45–54 years old*	2
*Over 55 years old*	3
Respondent nationality	
*Bangladesh*	11
*United States*	9
*United States/Canada*	1
*United States/Australia*	1
*Australia*	1
*Malaysia*	1
*United Kingdom*	1
*Japan*	1
Respondent place of employment during period working with Rohingya refugees	
*NGO*	25
*Multilateral organization (*e.g. *UN, WHO)*	1
Respondent position while working with Rohingya refugees	
*Physician*	12
*Nurse*	2
*Nurse midwife*	1
*Nurse practitioner*	2
*Clinical psychologist*	1
*Case manager*	3
*Paramedic*	1
*Other*	4
Period in which respondent worked with Rohingya refugees in Bangladesh^a^	
*2017*	14
*2018*	21
*2019*	10
*2020*	4

^a^Number of respondents known to be working with Rohingya refugees in that calendar year

Table 2 provides an overview of the key themes from 26 semi-structured interviews with health care professionals. These themes are discussed in greater detail in the below sections. Health care workers universally reported hearing accounts and seeing evidence of SGBV committed against Rohingya people of all genders by the Myanmar military and security forces. All health care workers interviewed observed some of the physical and psychological consequences of such acts against the Rohingya.

*“I would say everybody. I would absolutely say everybody we saw [was] suffering from the effects of the violence and from the trip altogether.... I don’t think we saw anybody in good condition.”* A nurse working in Cox’s Bazar in 2017

*“Trauma shows up in a lot of ways for a lot of different people. Everyone there is traumatized, I would say, without a doubt.”* A physician working in Cox’s Bazar in 2017 and 2018

**Table 2:** Overview of Findings

Theme	Sub-theme	Relevant Data
**Conflict-related Sexual Violence Experienced by the Rohingya in Myanmar**	Sexual violence accompanied by other violent acts	“*There was a woman in her probably late 40s but these women even in their late 40s looked like they were 70 and 80. She was there with her granddaughter. She had been raped and beaten and she saw her daughter and son killed. And the grandchild was the only one she had left.… And she was very disabled from the beatings, she could barely walk. Her back and her legs suffered severe injuries*.” A nurse working in Cox’s Bazar in 2017
	Men and women separated; killing the men, raping the women	*“When the Myanmar military would come to a village, they would immediately split the men and the women into two groups and they would pretty much immediately kill the men in front of the family and bury them in mass graves, and then the women would most likely be raped at that time and then either ... moved to a different section or ... out of that village.”* A nurse working at a primary health clinic in Kutupalong camp in 2018
	Sexual violence perpetrated by the Myanmar military, men in uniform, or police	*“It was the military … those who are associated with the army of that country.”* A psychosocial support officer and case manager working in Balukahli camp since 2018 *“About those who perpetrated them [the rapes], they said that [it was] a group of military people.”* A clinical psychologist working with Rohingya refugees since 2017 *“She started crying and talked to me about her experience of rape at the hands of the military, the Myanmar military.”* A nurse practitioner working at an outpatient clinic in Kutupalong camp in 2017
	Sexual violence committed by multiple perpetrators	*“Her age is between 42 to 45 years. The situation was the same, 25 militaries attacked their home at night all of a sudden. After the attack, the men of the house ran to the hill near the river, escaping through the kitchen door. After the men ran away, the woman was alone … her son was hiding in [the] poultry house. For that [her son hearing her gang rape while hiding] she is still ashamed and wishes to die. This woman is also a victim of gang rape by three military [men]. Her physical condition was so bad that it was beyond describable in words. Her uterus was very badly damaged. It used to bleed a lot after every few days. And she had a lot of pain.”* A paramedic and psychosocial support officer working in Cox’s Bazar since 2017
	Forced witnessing of acts of violence (including sexual violence) against family and community members	*“‘They [Myanmar military/security forces] took our young girls and they were raping them.’ They [Rohingya survivors] had seen and hear[d] probably this happening, and there was very little they could do about it.”* A volunteer physician working in Cox’s Bazar in 2018 *“When they were in Myanmar, they, a group of men who had uniform look like a police or army something like that, came to their house and … they were raped in front of their family.”* A nurse midwife working in Kutupalong camp in 2017
**Disclosure of Experiences of Sexual Violence**	Experience disclosed to health professional due to trust.	*Because I knew that there was a history of sexual violence among the population, I was alert to the possibility of that. And then I asked ... if she had had any problems with the military, or with anybody, when she was leaving Myanmar. And she hesitated a minute and then she started crying and said that she and I think around 14 other women had been taken and locked into a house, and that they were all gang raped. And some of them did not survive, but she was able to survive. She was now 40 years old.”* A nurse practitioner working at an outpatient clinic in Kutupalong camp in 2017
	History of sexual violence discovered during sexual and reproductive health, pregnancy-related care; sexual and reproductive health (SRH) and pregnancy-related care not often available for sexual violence survivors prior to arrival in Cox’s Bazar	*“I had a young woman.… She was probably in her early 20s or so. Came in with complaints of severe abdominal pain and vaginal discharge. Just on exam and everything else, it was quite certain that she had an STD, an STI of some sort and we did a quick pregnancy test and the pregnancy was.… She was very ill. She was most likely septic.… So we treated her with antibiotics and we watched her for the rest of the day.... She was in the clinic with us for about eight hours while we were taking care of her and during that time, first, she asked me if there was anything that could be done about the pregnancy. And this was not a common question in that camp, particularly. It’s not a culturally accepted option. So, we talked a little bit more and then I realized that the source of her question was she was raped by one of the Myanmar military personnel. That’s how she contracted the STI and also became pregnant.”* An emergency room physician working in Cox’s Bazar in December 2017 *“She was pregnant while she was visiting us in the clinic and she had been raped and she knew that this was a child of a soldier who she absolutely did not want to carry the baby….* *And she felt certain that she was going to be shunned and essentially cut off from all of her support network and she didn’t really want to keep the baby, but again, she had no options.”* An emergency nurse working in satellite health clinics in December 2017 *“One evening, my midwives came back with one nine months pregnant lady’s case and they were saying that she never had any antenatal check up.… what we revealed there was, she was raped nine months ago and just because it’s a matter of shame to disclose it within their community, or to come to the facility here, they kept her inside the house, she didn’t receive any antenatal check-up or anything. And now she is nine months pregnant, I mean, as a consequence of that rape.”* A medical officer at a health post in Camp 17 in 2018
	Observed increases in pregnancy and birth rates as a result of sexual violence	*“Of course, they had no way to check if they are pregnant or not and by the time they notice, it’s already 20 weeks and that was a time they come to the clinic. That was in November, because the crisis happened the end of August and most of the incidents happened the end of August up to beginning of September.”* A nurse midwife working in Kutupalong camp in 2017 “*Many of these women lost their husband and they were pregnant. [Health care workers asked] is it your husband’s child, do you think, or is this maybe a child of one of the rapes or something? None of them would talk to us about that at that point. None. They would not admit to being raped at all. It was such a stigma that no one was talking about that.”* A pediatrician working in Cox’s Bazar in 2017 and 2018 *“Basically anyone at that period of time that lived had a baby [was pregnant] ... that was the assumption. The assumption was that it was rape. In my trauma training, it’s not always in the best interest of the patient to ask them. So, I generally didn’t ask them if that was what had happened, because I was trying not to re-traumatize them.”* A physician working in Kutupalong camp in 2018
	Seeking pregnancy termination, complications from unsafe abortions and need for post-abortion care	*“[They had had] failed attempted terminations when they came.… [The staff] would call me because the patients were very unwell. At that point, obviously, we didn’t really discuss what had happened in terms of whether they were, who was responsible for that. We didn’t know, but we definitely saw a significant number of termination[s] … self-induced terminations of pregnancy when we were there”.* A physician working with Rohingya refugees in 2017 and 2018 *“The midwife came and said, ‘Madam, a patient ... has come, she wants [to have a] miscarriage, which means she does not want to keep the baby anymore … she … [wants to] have an abortion.... What can I say?.... When I started talking to that woman, she would burst into tears.... After asking her some questions deeply, like, ‘Why do you want to ruin the baby?’ She was told the Islamic thing that it is a great sin to ruin a baby. Then we would see that many became [emotional] and one or two [women were] saying that ‘I have a child; it is not mine and I ... I was not even married. I don’t want to keep this baby.’”* A clinical supervisor and health post manager in Balukahli camp since 2018
**Sexual Violence Experienced by Gender Diverse Patients**	Sexual violence against *hijra* and transgender people	*“Those who became victims among the gender diverse population [hijra, transgender], they became such victims even [in Myanmar] ...They were victims of different type[s] of teasing, they were victims of different types of harassment. But what they feared … the most [in Myanmar] was that they … [would be] killed…. Those groups [Rohingya ‘thugs’ in Bangladesh] used these people [coerced the hijra into sexual acts] [by threatening] to rape the sisters…[or] to rape his mother.”* A coordinator of services for male and transgender Rohingya survivors since 2018
**Mental Health Status of Survivors**	Evidence of depression and post-traumatic stress disorder (PTSD) in survivors	*“To make them talk, it takes time to get started and, of course, they cry. Also, they say I want to die, or I want to commit suicide, this kind of a thing.”* A nurse midwife working in Kutupalong camp in 2017
	Patients exhibiting trauma behaviorally and via oral narratives	*“I found her absent-minded. She didn’t look at me, didn’t talk, and only kept on crying. I observed different kind(s) of abnormal behavior.”* A paramedic and psychosocial support officer working in Cox’s Bazar since 2017 *“Most of the stories of trauma came from the patient’s mouth, like, ‘This is what happened to me,’ not necessarily ‘This is an injury, here are the scars.’ They said the trauma stories. I tried to have them express it as often as I could because my philosophy [is that for] patients who have gone through this, it’s part of the healing. We might be the only person that they might be able to narrate this story to. Whenever I had the opportunity or if I wasn’t as busy, I would try in a mindful way, just kind of ask the patient if they would be willing to share what happened…. I’m not a psychologist, I’m not a clinical psychiatrist, but I felt like I had to act like one for a lot of the patients because they’re coming in with vague symptoms like ... muscle aches or stomach pain. That’s sometimes somatization of trauma. That was an often thing that we saw as well.”* A volunteer physician working in Cox’s Bazar in 2018
	Psychosomatic manifestation of trauma	*“We try to identify the causes in medical science like blood loss or nutritional deficiency, it could be seen that those were not found. But … [if] she is feeling weak … or cannot concentrate in anything, then we would assume that it was a psychosomatic disorder and of course it was a mental disorder and surely they were suffering from some mental problem.”* A clinical supervisor and health post manager in Balukahli camp since 2018 *“[Rohingya patients were] very depressed, of course, and sometimes when the pregnancy is advanced ... so, people who cannot have abortion, they stay in the shelter until they deliver. I often got the call at middle of the night saying they are crying for abdominal pain or something like this. But ... I was pretty sure it was from PTSD, and because she didn’t have any sign during day, it was completely normal pregnancy, so I think it’s from mental.”* A volunteer physician working in Cox’s Bazar in 2018

### Conflict-related sexual violence experienced by the Rohingya in Myanmar

Health professionals noted a pattern of CRSV that included sexual violence and multiple perpetrator rape accompanied by other violent acts, such as beatings, shootings, and killing of family members.

*“Most of the cases are very similar. Families killed in front of them and raped, [survivors] escaped in the bush across the border.”* A nurse midwife working in Kutupalong camp in 2017

*“They give some example[s] like, when the army [was] at their villages and initially they [were] trying to beat them, and if there is any female … they raped them, and after rape, they killed them.”* A clinical psychologist working in Cox’s Bazar in 2018

*“She was raped by one of the Myanmar military personnel.”* An emergency room physician working in Kutupalong camp in December 2017

### Conflict-related sexual violence followed by other violent acts

Health care workers heard that their patients were forced to witness acts of violence, including sexual violence, against their family members. Women and men were separated during the attacks and respondents shared that after separation the men were killed and the women were forced to watch the men be killed, and then raped. Survivors shared that they left Myanmar for Bangladesh following these violent attacks.

*“Most of the women tell me [a] very, very similar story. When they were in Myanmar ... a group of men who had uniform[s] [which] look like a police or army, something like that, came to their house and ... [t]hey were raped in front of their family. After that, they took all the men.... They beat them and killed them, and they put all men into the fire and all women are taken to somewhere else in [an] empty house and they had to take off their clothes and they were naked. Men who ha[d] a kind of uniform look came to the house and they raped those women every day until they became unconscious. When those women [woke] up, [there was] fire around the house, so they tried to run away to the river which is a border to Bangladesh, and they crossed the river ... to come to Bangladesh.”* A nurse midwife working in Kutupalong camp in 2017

### Forced witnessing of sexual violence

Multiple patients shared stories with health care workers about being forced to witness the rape of others, including their mothers and sisters.

“*I remember an elderly lady who came in just with a bladder infection, a UTI. And then somehow the conversation devolved into the fact that she had watched her daughter-in-law get gang raped by six soldiers in their home*.” A volunteer physician working with Rohingya refugees in January 2018

*“Some of the patients who narrated, the male patients who narrated their loved ones having this [sexual violence] happen and witnessing it. There's no denial that this happened.”* A volunteer physician working in Cox's Bazar in 2018

This forced witnessing of sexual violence was often perpetrated against male survivors.

*“They had to leave their country all of a sudden, their houses were set on fire, their farming lands, their farming lands were set on fire, and even something like killing his brother by shooting in front of him or raping his sister in front him has also happened.”* A coordinator of services for male and transgender Rohingya survivors since 2018

### Rape by multiple perpetrators

Multiple health care workers heard from their patients about women being confined in houses where they were repeatedly raped, often by multiple perpetrators. Several respondents noted that survivors described the rape as being perpetrated in a systematic, organized fashion.

*“She and, I think, around 14 other women had been taken and locked into a house, and ... they were all gang raped.”* A nurse practitioner working in an outpatient clinic in 2017

Sexual violence was also perpetrated against young people one respondent shared the story of a young girl of about 12 or 13 years old.

*“There were five women in total who took shelter there, including her [the young girl’s] mother, elder sister, and aunt. Then four to five military officers attacked there. They blindfolded their eyes and tied up their hands and feet. Once they were tied up, they became victims of gang rape. After the gang rape, they decided to burn these women.... But how this teenage girl managed to untie her hands only Allah knows that. She freed her hands.... She fled away from there to a piece of land.... When the girl crawled into that land, she was shot.… Her mother and others were burnt. All of them. She saw that happening.”* A paramedic and psychosocial support officer working in Cox’s Bazar since 2017

### Sexual violence experienced by gender diverse people

Perpetration of sexual violence in Myanmar was not limited to women and girls – acts of sexual violence were also experienced by men, boys, *hijra* (third gender) and transgender people. Respondents shared stories of providing care to these patients, though these patients were often reluctant to share their experiences.

*“We have found ... adults and young boys, they also became victims of physical violence.... There are one or two who had become victims of sexual violence, there are one or two like this.”* A clinical psychologist working with Rohingya refugees since 2017

### Disclosure of experiences of sexual violence

Respondents shared that Rohingya women often shared incidents of sexual violence that they had experienced while receiving care for a variety of other health reasons, such as addressing sexual and reproductive health (SRH) complaints, seeking care for physical symptoms or mental health support, as opposed to encounters for post-rape care services. Health care providers also described how experiences of sexual violence were only shared after patients were asked directly during initial medical intake interviews.

*“She had complaints of vaginal discharge, so I asked her if I could examine her vaginally. And then, when I did the exam, it looked like she had some trauma, just from the scars on her perineum. So, I asked her a few questions about that, and then she started crying and talked to me about her experience of rape at the hands of the military, the Myanmar military.”* A nurse practitioner working at an outpatient clinic in Kutupalong camp in 2017

### Perceived increases in the number of pregnancies and births following sexual violence

Health care workers sometimes learned about the sexual violence their patients had experienced while women were seeking pregnancy-related care. Respondents noted observing higher numbers of pregnant women in the months that would correspond to conception during the period of violence in Myanmar, around August 2017.

*“It was … nine months after August of 2017. So, there was a huge increase in the births because of all the women who were raped.”* A physician working in Kutupalong camp in 2018

*“I expected we would have a lot of unwanted delivery and undesired babies in April [un]til June.”* A nurse midwife working in Kutupalong camp in 2017

A few respondents believed that many of the children who they saw in their health centers were born of rape.

*“The second time I went back [to the camps] in July [2018], that would have been ... 11 months [after the violence], so many of the babies that were products of rape had been born. It was untalked about. Nobody said anything and the babies would be brought in…. They'd be accepted into the community, into the family. Obviously, the mom was there, but there was no discussion of how that baby came into being.”* A pediatrician working in Cox’s Bazar in 2017 and 2018

### Seeking pregnancy termination

Some health care workers were specialists in SRH and described women asking to terminate their pregnancies which were reportedly related to rape prior to arrival in Bangladesh. It was dually challenging for survivors to cope with an unwanted pregnancy and seek termination of this pregnancy due to both cultural and religious norms and legal restrictions on abortion in Bangladesh.

*“They were interested in this [abortion]. They were interested because everyone in the family knew that she was unmarried. Moreover, she couldn’t tolerate the fact that she was going to be the mother of a child [born of rape].”* A paramedic and psychosocial support officer working in Cox's Bazar since 2017

### Mental health status of survivors

Health workers shared that many survivors who they treated showed signs of mental health conditions associated with trauma. Many patients were reluctant to share their experiences or express their feelings, demonstrating passivity during treatment, and often remaining silent and motionless.

*“We ... found that most of them [the refugees] were suffering from PTSD. They had full-blown symptoms. We noticed that ... [they] even didn’t feel comfortable in talking to us. We were taking care of them, but they couldn’t manage to trust us completely.... At that time, there were some symptoms in them like their response, anxiety, hastiness, I mean very unstable, and some were in depression, like they were depressed and were not talking at all.... And it was like they came through such a crisis, many of them seemed to be totally blank. No emotion was working on their mind. They couldn’t answer any question they were being asked. They cried continuously. Upon being asked about anything, the first answers they gave were about what they had seen there. How they have come here. They described the difficulties of the situation, how much they had to walk, so many deaths, then rapes and other fears. That they were scared, these things were very common in them.”* A clinical psychologist working with Rohingya refugees since 2017

Survivors also commonly presented to health care workers with psychosomatic complaints. Upon further probing, many of these complaints were presumed to stem from trauma.

*“A huge number of our patients had somatic complaints, like very vague. Like, ‘I've had a headache or a stomachache or backache.’ And then, when you dig deeper into how long have these things been going on, it's been going on as long as they left their home or as long as they've been living in the camps. So, we certainly saw a lot of patients who we couldn't find anything wrong with them clinically and it seemed like the providers would attribute it to just a stress reaction that's manifested in a physical way. And that was very, very common.”* An emergency nurse working in satellite health clinics in December 2017

### Barriers to disclosure of sexual violence

It was also noted that sometimes those who had witnessed sexual violence were more likely to disclose it than the survivors themselves. Health care workers shared that survivors were often reluctant to disclose due to stigma, which could further contribute to their trauma and also create an additional barrier to seeking care.

*“The men were the ones who were more likely to share their trauma stories, and also talk about the women having this happen to them. The women, I think, that I did have speak about their stories, they focused a lot on the environmental things, like the houses being burned, or I mentioned the babies being burned or thrown in the river. Those came from women that I spoke to. They didn't really go into the gender-based violence or the rape when they shared their trauma stories.”* A volunteer physician working in Cox’s Bazar in 2018

## Discussion

Health workers’ narratives provided new perspectives regarding the systematic and complex sexual violence inflicted upon the Rohingya population. Interviewing health professionals allows for the collection of individual stories, observation of population level trends as each professional has likely treated hundreds of Rohingya patients and analysis of the specific physical and psychological sequalae of sexual violence. This study further reinforces the importance of collecting data from sources other than the survivors themselves. By interviewing health professionals, this study enabled the collection of survivor and witness accounts more broadly, while avoiding retraumatizing survivors and protecting patient privacy.

This study contributes additional data to a growing body of literature documenting CRSV committed by Myanmar state forces against the Rohingya population in 2017 and its resulting impact on Rohingya survivors, their families and communities [8, 11, 12, 18]. This is one of the first studies relying on accounts of health professionals working in Bangladeshi refugee camps to describe human rights violations against the Rohingya. Importantly, it provides documentation related to two issues that were previously only reported sporadically: occurrences of forced witnessing and of excess births attributed to rape [19, 20].

Data collected from this study corroborates previous reports and contributes to the evidence that sexual violence against the Rohingya in Myanmar was widespread and followed common patterns of perpetration, it was perpetrated by members of the Myanmar military and others wearing uniforms, and, is consistent with and corroborates many other reports [2, 5, 6, 8, 10–12, 19].

The finding that survivors routinely disclosed experiences of sexual violence while seeking care for other health concerns, including acute injuries, pregnancy-related care, and psycho-social support, highlights the need to provide health professionals with appropriate training in how to ethically screen for sexual violence in high-risk populations. These findings align with research by Tay et al. (2019) and Parmer et al. (2019) which both indicated that Rohingya refugees were reluctant to report sexual violence or seek care and showed limited help seeking behaviors, likely due to past restrictions on rights and health care in Myanmar [21, 22]. These findings indicate the importance of providing cultural relevant care, potentially outside of traditional medical care delivery pathways, such as through community volunteers or integrated care provision models with other services [21, 23, 24]. Training on screening for CRSV should include skill building on trauma-informed manners of engaging with conflict-affected populations as part of routine medical care [25–27]. Data collected for this study suggests that such training would be beneficial for health professionals on both long-term and short-term assignments providing care for populations affected by conflict featuring CRSV.

Multiple narratives about forced witnessing of killings and rapes underscores the need to consider this violence and its impact in both the public health and human rights documentation and accountability spaces [13, 28, 29]. Such instances of witnessing CRSV, though they may not cause physical trauma, are associated with long-term mental health trauma. One study of elderly Austrians found that 18.2% of those who witnessed acts of sexual violence during World War II reported post-traumatic stress disorder (PTSD) many years after the conflict had ended [30]. Naher et al. (2020) found that the negative impact on interpersonal relationships, stemming from the witnessing of sexual violence, was a key issues mental health and psychosocial issue that Rohingya survivors faced [31]. Additionally, a survey of Rohingya adolescents by O’Connor et al. (2021) found that on average they had experienced 3.5 traumatic events, including sexual violence, compared to only witnessing 1.6 such events among Bangladeshi adolescents; this would seem to indicate a larger burden of witnessing among Rohingya survivors with important implications for long term mental health impacts and intergenerational trauma [18].

Despite clear evidence showing the deep impact of forced witnessing on the bystander, forced witnessing of sexual violence is not always articulated as an element of sexual violence or recognized as a separate violation occurring at the same time [30, 32]. Recognition of forced witnessing as an element of sexual violence or a connected trauma can provide deeper appreciation of the varied experiences of CRSV survivors and the crimes committed against their communities, and can promote efforts for redress, remedy, and rehabilitation.

This study also provided unique data on the not-often-openly-discussed topic of excess births – an observed increase in the number of births that would be expected based on historical data and clinical experience – resulting from rapes committed in Myanmar, and also on survivor interest in abortions. The estimations of increased pregnancy and birth rates shared by our respondents are consistent with published studies and observations by other organizations suggesting birth rates above the historical baseline among Rohingya refugees in Bangladesh [20, 22, 33]. Pregnancy termination was challenging for survivors, given the cultural and religious setting, stigma, underreporting of sexual violence and resulting pregnancy, very limited services for abortion despite some successfully implemented care models with limited reach, extreme complications from unsafe abortions, and legal restrictions [24, 34, 35]. The combination of an increased number of births and the difficulty in accessing safe pregnancy termination would seem to indicate that Rohingya women were sometimes forced to continue pregnancies that were unintended or unwanted. Forced pregnancy, in international criminal law and international humanitarian law, includes detainment or confinement as a way the crime of forced pregnancy is understood. The experience of Rohingya survivors being forced to continue pregnancies because of a lack of access to abortions provides an example of an additional way forced pregnancy could also be defined [36, 37].

### Limitations

Our study has several limitations. Respondents were recalling their experiences and specific patient histories from events as far back as 2017, though some respondents had relied on written notes from their time spent in Cox’s Bazar recall bias is inherent in the data presented. Some respondents did not begin seeing Rohingya patients until 2018 or later and could speak to the longer-term impacts of the violence experienced but were not able to comment directly on acute injuries sustained in Myanmar.

Study respondents came from a diversity of cultural and geographic backgrounds, but no Rohingya respondents were able to be included in the sample, therefore comments and observations about behaviors impacted by cultural norms should be viewed as highly contextual. The survivors’ experiences recounted in these interviews often relay conversations conducted through the help of an interpreter and therefore must be interpreted with note to the fact that they were communicated through multiple filters, both cultural and linguistic. In spite of these limitations, the narratives collected of Rohingya survivors experiences showed significant similarities.

As with all qualitative research, the analysis and interpretation of data is impacted by interpretation biases that are introduced by the research team. To address this potential bias the research team was multidisciplinary, culturally diverse, and worked collaboratively to mitigate potential biases in the interpretation of results.

### Implications for practice and future research

There are key implications of this research for both practice in response to CRSV in humanitarian and conflict-affected settings and for future research areas.

The findings of this research emphasize the importance of offering universal and comprehensive trauma-informed services to all refugees with the presumption of high rates of trauma in this population. Many survivors will never identify themselves and while some survivors may not have been the direct victims of sexual violence, forced witnessing constitutes an additional source of trauma that may not be identified or shared explicitly [18, 21, 35]. Survivor-centered and trauma-informed care training should be a required training component for all health care workers working in similar settings. This is important not only to enhance the care of survivors, but also as a means of addressing potential vicarious trauma affecting health workers. A survivor-centered approach also requires that clear referral pathways and critical mental health services be provided in a consistent and long-term manner and that there is integrated and complementary responses across the health sector [38]. In the presence of these resources, health screenings that include opportunities to sensitively engage survivors of sexual violence should be incorporated, given that many survivors revealed their experiences of sexual violence while seeking other health care. In the absence of mental health and SGBV services, screening for sexual violence is ethically complex and may be ill-advised [39–42].

Future research could involve multiple areas of inquiry. Continued exploration of the long-term impact of CRSV on Rohingya survivors and on their collective trauma is needed. We must also study the patterns and acute and long-term effects of forced witnessing. The impact of survivor trauma on health workers who care for them should be studied and analyzed. Finally, in uplifting the voices of health care workers, this study highlights the importance of increasing the use of alternative research methods for collecting sensitive data in a manner that avoids the re-traumatization of survivors.

## Conclusion

While this study focused on documenting clinical presentations of physical and psychological harm caused by past perpetration of conflict-related sexual violence by the Tatmadaw and Myanmar security forces, these groups continue to use sexual violence as a tactic of intimidation and terror elsewhere in Myanmar. Our findings can inform human rights investigations and service delivery in Rohingya refugee populations in Bangladesh and for ethnic minorities elsewhere in Myanmar. These findings can also support wider calls for justice and accountability for the violations perpetrated by the state forces of Myanmar, in the past, present, and future.

## Data Availability

The deidentified data used for this analysis can be made available upon reasonable request, in accordance with the study’s informed consent process. For further information, contact the corresponding author.

## Abbreviations

CRSV: Conflict-related sexual violence
PTSD: Post-traumatic stress disorder
SGBV: Sexual and gender-based violence
SRH: Sexual and reproductive health
UN: United Nations
